# Mobilizing Health Promotion Through Canada’s Student Mental Health Network: Concurrent, Mixed Methods Process Evaluation

**DOI:** 10.2196/58992

**Published:** 2025-02-27

**Authors:** Amy Ecclestone, Brooke Linden, Jessica Rose, Kiran Kullar

**Affiliations:** 1 School of Public Health Sciences, University of Waterloo, Waterloo, ON, Canada; 2 Health Services and Policy Research Institute, Queen’s University, 21 Arch Street, Kingston, ON, K7L 3J8, Canada, 1 3433336127; 3 Queen’s University, Kingston, ON, Canada

**Keywords:** mental health, health promotion, program evaluation, postsecondary, knowledge translation

## Abstract

**Background:**

Mental health issues among Canadian postsecondary students are prevalent. In tandem, an increased acknowledgment of the need for upstream mental health support has been highlighted. While the majority of institutions offer some form of mental health promotion, research suggests students are failing to access support due to barriers including lack of awareness, geographical and financial barriers, and lack of relevance in offerings. Canada’s Student Mental Health Network is a web-based knowledge mobilization initiative designed to fill these gaps. With content created and curated “for-students, by-students” and reviewed by subject matter experts, the Network serves as a one-stop shop for evidence-based, mental health support for postsecondary students.

**Objective:**

The goal of this research was to conduct the first component of a comprehensive program evaluation of the Network. This paper details a formative, process evaluation after approximately 1 year of operations, with the goal of assessing acceptability and feasibility.

**Methods:**

Using a concurrent mixed methods study design, quantitative and qualitative data were simultaneously collected from students in order to evaluate the acceptability and feasibility of the Network as a mental health promotion resource. Quantitative data were automatically collected through Google Analytics via the website over the course of the first year of operations. Data collected included the number of users accessing the website, user engagement, and user “stickiness.” Quantitative data were used to evaluate both accessibility and feasibility. Qualitative data were collected via individual, digital interviews conducted with a modest sample of students (n=8) across areas and levels of study. Qualitative data derived more detailed insights into user experience and website attributes, as well as feedback on content delivery, providing evidence used to evaluate feasibility.

**Results:**

A total of 1200 users globally accessed the Network within the first year of operations, with Canadian users accounting for nearly 90% of total website traffic. An overall 66% engagement rate was observed, with the average user visiting 7 pages per session. Further support for the acceptability of the Network is demonstrated in the Canada-wide reach of the content development and review team. Evidence for the feasibility of the Network was observed through website use statistics indicating the most frequently viewed pages aligned with our goals: providing mental health education and increasing awareness of available resources. Qualitative feedback provided additional context surrounding the feasibility of the space, including positive feedback on the esthetics, relevance, usability, inclusion, and accessibility. Areas for content expansion and improvements to accessibility were also identified.

**Conclusions:**

The results of this study provide evidence in support of the feasibility and acceptability of the Network as a web-based knowledge mobilization initiative in support of postsecondary students’ mental health and well-being. Future research will pursue a summative, impact assessment to evaluate utility.

## Introduction

Prevalence estimates for mental health–related problems, including above-average stress, psychological distress, and symptoms of mental illnesses, have increased significantly among Canadian postsecondary students over the past several years [1]. Indeed, data collected from Canadian postsecondary institutions via the 2022 National College Health Assessment III survey (n=11,322) revealed large proportions of students reporting moderate (47%) to high (37%) stress levels within the past 30-day period, with over half (52%) reporting their stress levels to have impacted their academic performance [2]. Additionally, approximately one-third (33%) of respondents screened positive for serious psychological distress, while many reported having received a diagnosis for anxiety (23%) or depression (25%) [2].

Importantly, excessive stress has been linked to a number of negative academic (eg, decreased performance and decreased motivation) and health (eg, anxiety and depression) outcomes for postsecondary students [3,4]. In recent years, a more in-depth exploration of the sources of student stress has been conducted in the development and deployment of the Postsecondary Student Stressors Index. This work has revealed that stressors experienced by students within the postsecondary setting reach beyond academics, also spanning the learning environment, campus culture, personal, and interpersonal domains [5,6]. Importantly, this suggests a need for holistic mental health promotion support, including providing resources that extend beyond the academic realm. Further complicating things, postsecondary-aged Canadians were also the age group identified as the most likely to report dramatically declining mental health throughout the COVID-19 pandemic [7]. The long-term mental health fallout of the pandemic continues to be unknown, further reinforcing the need to provide well-being support to students as they navigate the stressors associated with the postsecondary setting.

Strategies to address and support postsecondary mental health have emerged at the national, provincial, and institutional levels [8]. At the postsecondary level, many institutions have attempted to improve student mental health by offering both upstream and downstream mental health services [8,9]. Upstream services aim to intervene prior to the development of symptoms of mental illness (eg, mental health promotion and mental illness prevention), while downstream interventions (eg, counseling and pharmacological therapy) aim to treat individuals who have already reached a clinical threshold. These service levels align with Keyes’ seminal conceptual framework, the dual continuum of mental health and mental illness, which conceptualizes mental health and mental illness as existing on interrelated, but separate continuums [10]. A student who is experiencing a period of poor mental health, but who has not yet reached the clinical threshold for a mental illness, may benefit greatly from the use of upstream services. Unfortunately, the majority of students experiencing a decline in their mental health seek downstream treatments as an initial solution. As a result, the demand for downstream campus mental health services (eg, counseling) over the past decade has far outpaced institutions’ capacities to deliver timely care [11]. Placing additional focus on upstream mental health promotion and mental illness prevention resources may contribute toward alleviating the current bottleneck observed at the treatment level by encouraging students to implement appropriate forms of help-seeking that align with their level of need. Bolstering upstream supports, including mental health promotion, may also improve students’ resilience and education regarding adaptive and effective coping mechanisms, empowering students to better manage stressful situations.

While some form of mental health promotion is offered on most postsecondary campuses, a national review of mental health and well-being services on Canadian postsecondary campuses found that only 70% of respondents felt students were well-informed about available resources on campus, and about mental health issues in general [12]. Notably, many respondents (including campus service providers and institution administrators; 84%) also indicated they felt there was room for improvement in the mental health promotion efforts currently offered on their campus [12]. Further, mental health promotion activities were highly varied, with some offering programs aimed to inform students about available campus mental health services, reduce stigma, and educate students about mental illness. Other institutions focused on outreach, aiming to encourage students to seek help when needed. In many cases, the mental health supports offered were dependent on the resources (eg, financial, logistic, and staffing) available to the institution, with smaller institutions unable to provide as much support as their larger counterparts [12].

Together, these gaps and inconsistencies suggest a need for a comprehensive and holistic approach to mental health promotion, where resources are universally accessible to postsecondary students across Canada. With the overall aim of filling these gaps, Canada’s Student Mental Health Network (“the Network”) was created—a web-based, “one-stop shop” for mental health education and evidence-based resources. Content is tailored specifically to postsecondary students and is provided in a centralized, universally accessible location, focusing on three primary pillars of action: improving mental health literacy, encouraging the development of strong social support networks, and improving understanding of appropriate help-seeking and awareness of available resources.

A comprehensive evaluation of the Network, including formative and summative components, has been detailed and published elsewhere [13]. The purpose of this paper is to present the results of the formative, process evaluation of the Network after approximately 1 year of operation. Process evaluations are intended to assess 2 key components of program function: (1) whether the program is reaching the intended targeted population (acceptability), and (2) whether its delivery and function are consistent with program design intentions (feasibility) [14]. The key objectives of this formative evaluation were to determine the feasibility and acceptability of the Network to date and to inform the development of the protocol for the subsequent summative impact assessment.

## Methods

### Study Design

**Figure 1.**
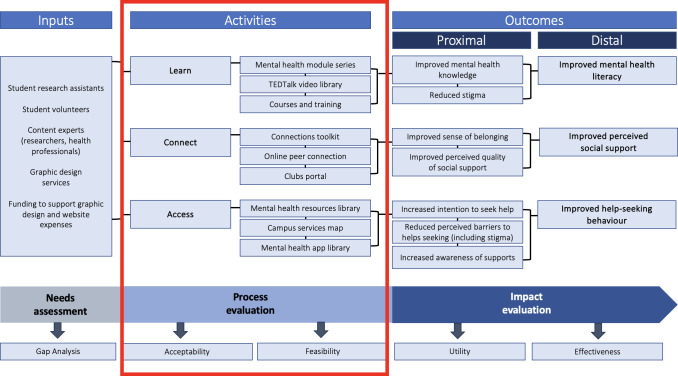
Logic model depicting program theory for the Student Mental Health Network, with components relevant to the formative process evaluation highlighted.

We used a concurrent mixed methods evaluation study design, combining both quantitative and qualitative data to gain more in-depth insights into the acceptability and feasibility of the Network. Best practices in evaluation dictate that the chosen evaluation design should align with the maturity of the initiative. Given the age of the Network at the time of this study, a formative process evaluation design was chosen, with a focus laid on process indicators selected to provide information about feasibility and acceptability, rather than focusing on outcomes evaluating impact. A future summative impact evaluation is planned for when the initiative has matured appropriately [13]. Figure 1 displays the logic model used to visually depict the program theory supporting the process evaluation.

### Initiative

The Student Mental Health Network is a website designed to be a web-based resource hub tailored toward postsecondary students, featuring mental health education and evidence-based tools and resources.

The target population for the Network itself is postsecondary students enrolled across Canada.

Centered around improving mental health literacy, encouraging the development of strong social support networks, and improving help-seeking behaviors, the content is intended to be relevant for all students, regardless of the type of institution attended (eg, university, college, and institute), years, areas, and levels of study. We have also aimed to develop content that is accessible, psychologically safe, and culturally competent for various subgroups of students who may be best supported by specific content areas, including medical students, members of the LGBTQ2S+ community, BIPOC students, Indigenous students, international students, and first-generation students. All content is created and curated by students and reviewed for validity by subject matter experts (eg, mental health researchers, clinicians, and medical professionals) using a collaborative, knowledge mobilization approach. Further information on the resources featured in each of the three content pillars (learn, connect, and access) has been published elsewhere [13].

### Recruitment

To meet the objectives of this evaluation, data were collected using two methods: (1) quantitative website use statistics, collected through Google Analytics, and (2) qualitative data collected through individual, cognitive interviews.

To collect information regarding the reach and coverage of the Network in terms of website engagement and user experience, we used quantitative website metrics collected passively through Google Analytics. These metrics included: the total number of users, average number of users (weekly, monthly, and annually), average engagement time (time spent on each component of the website) and user “stickiness” (average length of visits), page views, event counts (clicks, file downloads), and user region. The definitions of these specific criteria per the Google Analytics glossary can be viewed in Multimedia Appendix 1.

Second, individual, cognitive interviews were conducted using a “think aloud” technique to further evaluate user experience and highlight gaps in the Network’s delivery [15]. Participants were recruited using a convenience sampling method; a recruitment notice was posted on the Network website, as well as through the Network’s social media channels. Participants had to be computer literate and currently enrolled at a postsecondary institution at the time of the interview. A total of 8 interviews were conducted before data saturation was reached (eg, the point at which additional interviews were not producing new information and we were beginning to hear repetitive answers to interview questions). Table 1 shows the demographic characteristics of the 8 interviewees. In terms of procedure, participants were asked to screen share over Zoom (Zoom Video Communications Inc) as they navigated the Network website and talked the interviewer through their exploration, identifying content-related issues or preferences, as well as any navigational challenges. The interviews included questions related to important website attributes (eg, appearance, content, interactivity, inclusivity or representation, and accessibility) and perceived acceptability and feasibility of the space. Interviews were audio recorded and automatically transcribed, with field notes taken throughout the interview and immediately reviewed afterward.

**Table 1.** Descriptive statistics for demographics characteristics of cognitive interview sample.

Characteristic	Value (N=8), n (%)	
**Gender**		
Woman	5 (60)	
Man	3 (40)	
**Year of study**		
Y1 undergraduate	1 (13)	
Y2 undergraduate	2 (25)	
Y3 undergraduate	2 (25)	
Y4 undergraduate	2 (25)	
Graduate	1 (13)	
**Area of study**		
Health sciences	3 (38)	
Science	3 (38)	
Arts	1 (13)	
Graduate studies	1 (13)	
**Institution**		
Queen’s University	5 (63)	
Western University	1 (13)	
University of Toronto	2 (25)	

### Data Analysis

Descriptive statistics provided through Google Analytics were displayed as frequency tables. Cognitive interview data were analyzed through the use of the website attribute codes displayed in Table 2. Field notes were taken electronically during the interviews and reviewed immediately afterward. Website attribute codes were applied to relevant transcript areas. In the event that a new issue arose that was not captured by existing problem codes, a new code was created.

**Table 2.** Website attribute codes used to categorize cognitive interview responses and appropriate corrective actions to be taken.

Website attribute	Description	Corrective action
Esthetics	Comments on the look and feel of the website, including whether the color scheme, images, and multimedia used are appropriate.	Improve esthetics (additional graphics, multimedia, color)
Relevance	Comments on whether the content displayed on the website is not relevant to postsecondary student stress or mental health.	Add additional relevant content Remove irrelevant content
Accessibility	Comments on the accessibility of content is adequate (eg, ease of use for students with visual, audio, or other impairments).	Improve accessibility features of the website
Inclusion	Comments on whether the content provided adequately caters to a wide variety of students and important subgroups.	Change in wording Change the order of items
Usability	Comments on ease of navigation of the website, whether the website renders on mobile and web browsers adequately, etc.	Improve navigation Improve optimization

### Ethical Considerations

This research study was reviewed and approved for ethical compliance by the Queen’s University Health Sciences and Affiliated Teaching Hospitals Research Ethics Board (TRAQ #6041988). Google Analytics data were collected passively from website users and were not connected to any identifying information. Therefore, there was no consent component associated with this part of the study. Interview participants provided verbal, informed consent to participate (including audio and video recording of the interview) after reviewing the letter of information for the study. All qualitative data (eg, transcripts and extracted quotations) were deidentified for analyses. Interview recordings were kept confidential and stored as password-protected electronic files by the principal investigator (BL) as the data custodian. Interview participants were compensated with a CAD $20 (US $14.04) e-gift card to Amazon as thanks for their participation.

## Results

### Acceptability

Recall that acceptability refers to whether or not the program or initiative is reaching its intended targeted population [14]. Website use statistics were extracted from Google Analytics for the dates of May 1, 2022, to June 1, 2023, inclusive. A total of 1200 users globally accessed the Network within this time frame, where Canadian users accounted for nearly 90% of total website traffic (Multimedia Appendix 1). During this time frame, about 1500 “engaged sessions” were recorded, defined as the number of sessions (or visits) on the website that exceeded 10 seconds in length or had ≥2 page views. Users also spent some time navigating the Network, with the average user visiting approximately 7 pages per session. This resulted in an overall 66% engagement rate during the first year of operations. This data suggests that users are not only visiting the website but are spending time exploring its resources. Similarly, during this time frame, a total of 21,532 “events” (a specific interaction or occurrence on a website, such as a page view, click, or download) were recorded. Although we were not able to capture the age of these users, the continued and increased presence of users from across regions of Canada over time provides preliminary evidence in support of acceptability.

**Figure 2.**
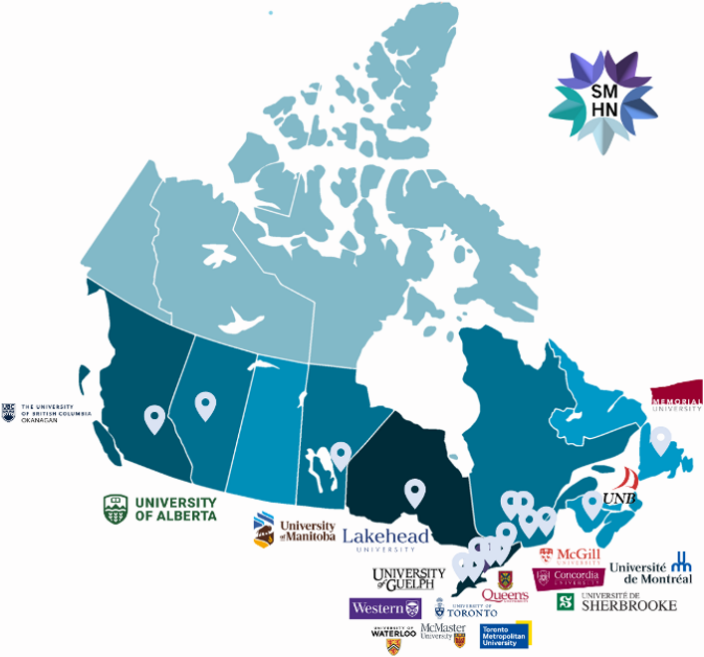
Map depicting the cross-Canada reach of the Student Mental Health Network after approximately 1 year of operations via the postsecondary institutions at which both student contributors and subject matter experts reside.

The acceptability of the Network is also reflected in the Canada-wide reach demonstrated through our content development team. Our student volunteers, who develop content and promote the Network, attend postsecondary institutions across the country and are enrolled across various programs, levels, and areas of study, including medicine, health science, political science, computer science, public health, education, and more. In addition to contributing students, we have a team of subject matter experts who serve as expert reviewers of website content to ensure its validity and reliability. These experts include faculty specializing in related research, Student Wellness Services staff and clinicians, and family physicians. Figure 2 shows the Canada-wide reach of the Network, including each of these contributing groups after 1 year of operations.

### Feasibility

Feasibility is determined by whether a program’s delivery and function are consistent with program design intentions [14]. We first gained insight into the feasibility of the Network by examining the website use statistics to determine which pages were viewed most frequently (Multimedia Appendix 1). In line with our goals, resources from the learn section of the Network were some of the most sought after the first year of operations (eg, module series, toolkits, and online courses). This was consistent with our initial needs assessment, which suggested that students were eager to improve their mental health education. Notably, several resources within the access pillar of the website were also within the most frequently visited pages (eg, campus services map, mental health resources bank, stress management resources, and mental health app library). This is consistent with our goals to encourage student awareness of available mental health resources. Finally, resources within the connect pillar of the website (eg, clubs portal and peer connection resources) were also among the most frequently visited pages, consistent with our goal to encourage the development of strong social support networks.

We also derived evidence for feasibility by conducting cognitive interviews with 8 students over the course of the evaluation period. Interviewees were asked to navigate through the Network website and comment on the appearance, relevance, accessibility, and usability. Overall, feedback was positive across interviews, with participants sharing that the site was visually appealing, easy to access, and relevant to them or their peers. Table 3 depicts the results of the interviews, as well as assigned website attribute codes.

**Table 3.** Responses to website attributes as indicated by interview participants (N=8).

Interviewee	Esthetics	Relevance	Accessibility	Inclusion	Usability
001	+^a^	+	+, x^b^	+	+
002	+	+	+	+	+
003	+, x	+	+	+	+, x
004	+	+	+	+	x
005	+	+	+	+	+
006	+	+	+, x	+	+, x
007	+, x	+	+	+	+
008	+	+	+	+	+

a + indicates a positive response to this attribute.

b x indicates that suggestions for improvement were recommended by the participant.

### Esthetics

When asked about the appearance of the website, participants indicated liking the color scheme and its consistency. The use of multimedia (eg, images, graphics, and videos) was reported to increase the visual appeal of the website. Participants also found that the content was displayed in an engaging way. In particular, they reported enjoying the format in which the toolkits and modules were presented, especially the integration of different types of media (eg, links to web pages, TED Talks, and worksheets). Participants indicated that the resources displayed as interactive multimedia were most esthetically pleasing (as well as easiest to navigate), suggesting that resources currently provided as downloadable documents be adapted to be displayed in a more esthetic, interactive alterative format.

### Usability

With respect to usability, participants noted that the website was easy to navigate, with clear pathways to associated resources. Participants appreciated the list of included items at the bottom of each page, clearly indicating each subsection of website pillars. Participants were also asked which type of device they used to access the online space, and whether the website and its resources rendered properly on their device. Despite the type of device and operating system used, the website was rendered properly. Many respondents suggested that a mobile app or version be developed specifically with tablets and mobile devices in mind.

### Relevance

When asked about the relevance of the website content to the student target population, all participants agreed that the content was relevant and valuable to a postsecondary audience. Participants also shared that it was clear content was intended for students from all schools and provinces and did not appear to be preferential to a single region. When asked if they would recommend the site or any of its resources to a friend or family member, every participant said yes, with some indicating that they had already done so.

### Accessibility and Inclusivity

Interview participants were asked to share their impressions of the accessibility and inclusivity of the website. Respondents shared positive comments regarding the accessibility of the site, stating that content appeared to be directed toward a wide range of student backgrounds and not targeted to any specific group. Participants also indicated that they were able to find everything they were looking for with ease, suggesting the website was user-friendly in terms of accessibility. Regarding inclusivity, participants thought the website content was generally applicable to all students, the more specific resources provided (eg, graduate students’ module, Indigenous well-being module, and intersectionality module) were especially inclusive as they targeted specific struggles experienced by students belonging to those groups. Respondents encouraged the development of additional resources (eg, modules and toolkits) targeting other specialty populations to enhance the inclusivity of the content. Overall, positive feedback was provided regarding both the accessibility and inclusivity of the website. Participants’ recommendations regarding additional resources and content that could be improved or enhanced are displayed in Table 4.

**Table 4.** Recommendations provided by interview participants (N=8) for additional or adapted content for the Student Mental Health Network.

Area of website	Suggested addition or update
Learn	A library of recommended self-help books fostering positive mental health, resilience, and well-being Development of an international student module, LGBTQ+^a^ module, and expansion of resources for students of color, LGBTQ+ students, international students, and Indigenous students
Connect	A library of social media accounts that aim to promote mental health
Access	Reformatting of the Mental Health Resources bank to be displayed similarly to the clubs bank
General	A newsletter updating interested students on new resources added to the site, and on general student mental health news (eg, recent impactful research) A Q&A^b^ page for the site to answer any common questions students may have while navigating our site Option to increase text size Option to select “read aloud” or prerecorded audio for some sections and resources French translation of website content Development of a mobile-optimized version of the Network

a LGBTQ+: lesbian, gay, bisexual, transgender, queer, and other sexual and gender minority.

b Q&A: question and answer.

## Discussion

### Principal Findings

As prevalence estimates for mental health issues have increased over the past decade [1], increasing attention has been paid to postsecondary mental health. The development of institutional frameworks and guiding documents such as the Okanagan Charter [16] and Canada’s National Standard for the Psychological Safety and Wellbeing of Postsecondary Students [17] have emerged, alongside new forms of data collection such as the Canadian Campus Wellbeing Survey [18]. Across the board, the recommendation for improvements in the targeting of mental health support for students is evident [8]. With the demand for downstream treatment-based services (eg, counseling and medication) continually surpassing the campus’ capacities to provide care to all, there is a glaring need to work upstream. Bolstering and improving the targeting of these upstream mental health supports is imperative to reducing the burden on downstream campus mental health services by empowering students to improve their resiliency and develop a toolkit of resources to turn to during times of increased stress where lower-intensity support may result in marked improvements.

The Student Mental Health Network offers 1 such solution as a web-based mental health promotion resource specifically targeted toward postsecondary students and universally accessible across Canada. The Network provides unlimited access to mental health education and resources, addressing many of the common barriers cited by students attempting to access mental health supports, including financial restrictions, lengthy wait times, stigma, and logistical access (eg, time, location, or travel-based constraints) [19]. The findings from this formative process evaluation have provided preliminary evidence for both the acceptability and feasibility of the Network. Results showed the imitative is reaching its intended target population (acceptability), and that its delivery and function are consistent with program design intentions (feasibility). In addition to positive feedback from the target population, opportunities for growth were also identified.

Results from Google Analytics provided evidence for accessibility (that the initiative is reaching its intended targeted population), with web use statistics indicating that a total of 1200 users globally accessed the Network within the first year of operations, the vast majority of whom resided in Canada (90%). This data suggests that the Network is reaching its intended target population. Providing additional support to this conclusion is the Canada-wide reach of the Network, evidenced through the regional distribution of our content contributors and expert reviewers, including postsecondary students, mental health researchers, clinicians, and physicians from across the country. Users also spent time navigating the Network, with the average user visiting approximately 7 pages per session. This finding provides evidence that users are not simply popping in and out of the space, but rather, are taking the time to view multiple pages and absorb some of the information provided. Overall, the Network reported a high engagement rate (66%) within its first year of operations. Overall, this data suggests that users are not only visiting the website but are spending time exploring its resources, as we hoped they would. Collecting passive user data via Google Analytics allowed us to cast a much wider net with respect to determining usership and avoid issues common to survey-based research, such as low response rates and selection bias.

Feasibility was supported by Google Analytics data in addition to the data derived from cognitive interviews. Recall that the goal of the Network was to provide Canadian postsecondary students with a web-based, “one-stop shop” for mental health education and evidence-based resources focusing on mental health education, social support, and improving awareness of available mental health resources. After the first year of operations, results suggest that the Network’s delivery and function have been consistent with our goals. Valid and reliable content has been created across all 3 areas of focus and is well-received by students. Data from Google Analytics showed that resources within all 3 sections fell within the top 20 most frequently visited pages of the Network, with resources in the learn and access sections being the most popular. Results from interviews further supported students’ endorsement of the Network and its resources, with positive reviews regarding website esthetics, relevance of content, accessibility, inclusion, and usability. Interviewees identified some areas for improvement, including additional content to fill some existing gaps, as well as minor esthetic or display changes to existing resources, but no resource was identified for removal or irrelevance. Here, we see the added value of the mixed methods design of the evaluation, where valuable contextual information was provided as a result of the cognitive interviews compared to what would have been gleaned from the collection of quantitative data alone. These interviews allowed for a detailed walkthrough of the website, facilitating a rich conversation about numerous elements (eg, relevance, accessibility, and esthetics). Overall, the design elements highlighted by study participants echo those identified by Garret et al [20] as key to thoughtful website design and driving user engagement.

### Limitations

Despite its strengths, there are several limitations to this research that should also be acknowledged. While the Network is a novel initiative aiming to fill important gaps, its novelty also presents evaluation challenges given the absence of a standardized process for evaluating this type of initiative. Despite this limitation, we did our best to thoughtfully design a comprehensive program theory and evaluation plan, including both the process evaluation outlined here, as well as a future summative impact evaluation. In terms of data collection, only 8 interviews were conducted with all students attending postsecondary institutions in Ontario, resulting in limited geographic variability in our qualitative sample. However, we did see good variability in terms of other characteristics, such as gender, area, and level of study. Furthermore, while our qualitative sample was small, we did find that we reached data saturation in our interviews. Additionally, data collected through Google Analytics were able to provide us with information on the number of people visiting the website over time and from which regions, but not the age groups to which those visitors belonged. As a result, we can conclude that website traffic increased over time, but we do not have quantitative evidence to confirm that those visitors were postsecondary-aged individuals. Additionally, while the Network has demonstrated a cross-Canada reach after the first year of operations, there is an uneven distribution of users and content contributors with the majority situated in Ontario. This may be due to the fact that the Network was initially created by a group of students at a single, Eastern Ontario university, or due to the vast majority of postsecondary institutions in Canada being located in this province. Moving forward, we plan to continue working on extending the reach of the Network across the country. Finally, website content is currently only available in English, which poses an accessibility challenge for French-speaking postsecondary students. Going forward with Network development, we aim to translate content into French to provide equitable access to mental health resources to Canadian students in both official languages.

### Conclusions

Bolstering upstream supports like mental health promotion may help alleviate the current bottleneck observed at the downstream service level on postsecondary campuses. Canada’s Student Mental Health Network aims to contribute toward alleviating this issue by delivering a novel, innovative method of providing universally accessible, evidence-based mental health promotion to postsecondary students across the country. The Network provides a centralized location for mental health education and resources, created for-students, by-students, and validated by subject matter experts, including physicians, clinicians, and mental health researchers. The space will contribute to breaking down common barriers to help-seeking, such as geographic restraints (eg, by providing universally accessible content that can serve online students, as well as those with travel or logistical obstacles), financial restraints (eg, by providing cost-free education and resources wherever possible), and lack of awareness (eg, by maintaining an active social media presence and partnering with Student Wellness Services at postsecondary institutions across Canada to bolster promotion). The participatory, for-students, by-students approach is a key strength of the Network and a characteristic that many students have highlighted as a unique and attractive quality of the space. The Network is a first-of-its-kind initiative that aims to fill gaps in current mental health promotion resources provided to students using an innovative, collaborative design. Through the provision of relevant and focused content and the facilitation of social connection, the Network will support and empower students to take ownership of their mental health and well-being by providing them with the toolbox they need to build and maintain resilience.

The formative process evaluation component for the Network has now been completed, with data collection for the impact evaluation currently underway. The next steps will include the ongoing creation, curation, and refreshing of the Network resources in order to meet the changing needs of the target population, as well as to fill any gaps identified throughout the evaluation process. Resources will continue to be updated to ensure a high level of accessibility and relevance. Preliminary results from this evaluation show the Network is meeting its preliminary objectives. The summative assessment of the Network will aim to further evaluate these objectives and provide additional evidence of overall impact.

## Supplementary material

10.2196/58992 Multimedia Appendix 1 Google Analytics terms glossary and website usage statistics.
