# Supplementary material for: Mobilizing Health Promotion Through Canada’s Student Mental Health Network: Concurrent, Mixed Methods Process Evaluation
Source: JMIR Form Res. 2025 Feb 27;9:e58992. doi: 10.2196/58992 (PMC11884303; doi:10.2196/58992)

**Multimedia Appendix A**

*Table A-1. Google Analytics Glossary Definitions for Relevant Metrics*

| **Term** | **Definition** |
| --- | --- |
| Metric | A quantitative measurement, such as an average, ratio, percentage, etc. |
| Number of users | The number of individuals accessing your website or app. Reported as a total as well as a weekly, monthly, and annual average. |
| Engagement | Engagement is any user interaction with your site or app. |
| User Activity over time | User activity over time shows the number of people who used your website or app in the past 30 days, 7 days, and 1 day. […] includes only users who engaged with your website while the site was in focus. |
| Average engagement time | The average amount of time a user engages with your website or app. |
| Session | A session is a group of user interactions with your website or app that take place within a given time frame. In Analytics, a session initiates when a user either […] views a page or screen. An **engaged session** is a session that lasts longer than 10 seconds or has at least 2 pageviews or screen views. |
| Events | An event allows you to measure a specific interaction or occurrence on your website or app. For example, when someone loads a page, clicks a link, or completes a purchase. |
| User Stickiness | User stickiness compares engagement by active users over a narrower time frame with their engagement over a broader time frame. The ratios can help you understand how well you retain users over time — higher ratios suggest good engagement and user retention. |
| User Region | The geographic location from which users are accessing your website or app. |

*Figure A-1. Users by Country and Region of Canada (May 1, 2022 - Jun 1, 2023)*


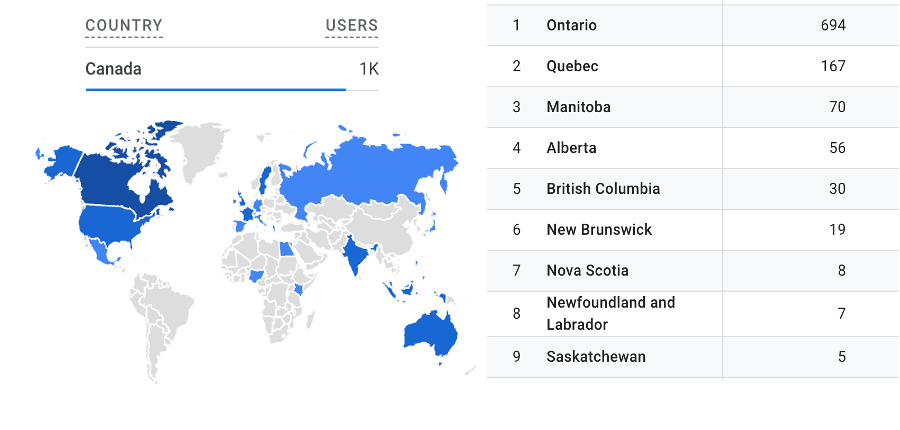


*Figure A-2. Website Usage and Engagement Statistics for Network (Top 20)*


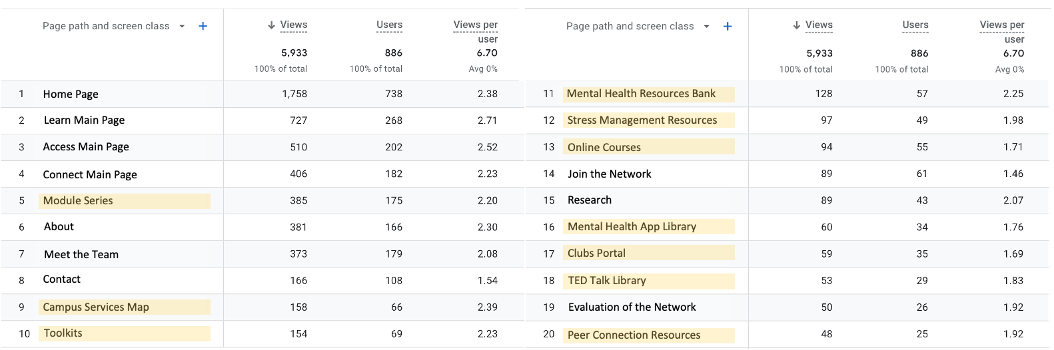

Supplement: Multimedia Appendix 1 [file formative-v9-e58992-s001.docx]
